# Bone morphogenetic protein 7 mediates stem cells migration and angiogenesis: therapeutic potential for endogenous pulp regeneration

**DOI:** 10.1038/s41368-022-00188-y

**Published:** 2022-07-20

**Authors:** Cheng Liang, Qingqing Liang, Xun Xu, Xiaojing Liu, Xin Gao, Maojiao Li, Jian Yang, Xiaotao Xing, Haisen Huang, Qi Tang, Li Liao, Weidong Tian

**Affiliations:** State Key Laboratory of Oral Diseases & National Clinical Research Center for Oral Diseases & Engineering Research Center of Oral Translational Medicine, Ministry of Education & National Engineering Laboratory for Oral Regenerative Medicine, West China Hospital of Stomatology, Sichuan University, Chengdu, China

**Keywords:** Mesenchymal stem cells, Regeneration, Stem-cell differentiation

## Abstract

Pulp loss is accompanied by the functional impairment of defense, sensory, and nutrition supply. The approach based on endogenous stem cells is a potential strategy for pulp regeneration. However, endogenous stem cell sources, exogenous regenerative signals, and neovascularization are major difficulties for pulp regeneration based on endogenous stem cells. Therefore, the purpose of our research is to seek an effective cytokines delivery strategy and bioactive materials to reestablish an ideal regenerative microenvironment for pulp regeneration. In in vitro study, we investigated the effects of Wnt3a, transforming growth factor-beta 1, and bone morphogenetic protein 7 (BMP7) on human dental pulp stem cells (h-DPSCs) and human umbilical vein endothelial cells. 2D and 3D culture systems based on collagen gel, matrigel, and gelatin methacryloyl were fabricated to evaluate the morphology and viability of h-DPSCs. In in vivo study, an ectopic nude mouse model and an in situ beagle dog model were established to investigate the possibility of pulp regeneration by implanting collagen gel loading BMP7. We concluded that BMP7 promoted the migration and odontogenic differentiation of h-DPSCs and vessel formation. Collagen gel maintained the cell adhesion, cell spreading, and cell viability of h-DPSCs in 2D or 3D culture. The transplantation of collagen gel loading BMP7 induced vascularized pulp-like tissue regeneration in vivo. The injectable approach based on collagen gel loading BMP7 might exert promising therapeutic application in endogenous pulp regeneration.

## Abstract (web summary)

BMP7 as a regenerative signaling molecule mediates stem cell migration and odontoblastic differentiation (a) and as a pro-angiogenic factor promotes revascularization of endothelial cells (b). Collagen gel supports cell adhesion, spreading, and viability (c).

## Graphical abstract

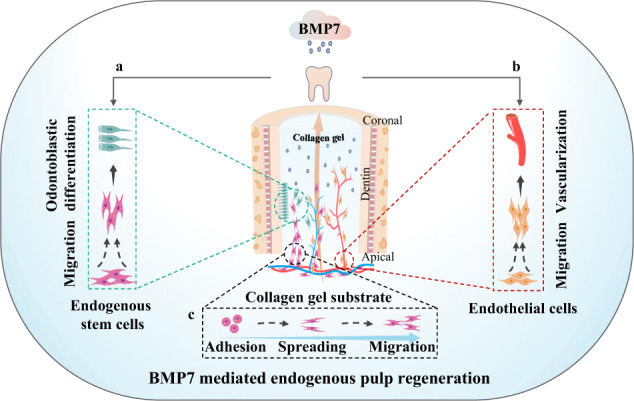

## Introduction

Dental tissue damage and loss impair oral and general health. Recently, the tissue engineering strategy has been introduced for dental tissue regeneration or bone regeneration.^1,2^ Many studies have been conducted in the fields of pulp-dentin regeneration and periodontal tissue regeneration. Root canal therapy (RCT) removes the dental pulp suffering severe infection in the mature teeth, which would make the teeth lose nutrition and become fragile.^3^ Regenerative endodontics procedures (REPs) such as revascularization promote the continued growth of immature roots but resulted in restorative responses rather than physiological pulp tissue regeneration.^4^ There are growing appeals for pulp regeneration strategy in the clinic.^5^

Stem cells residing in the dental tissues share the self-renewal and differentiation capacities, which provides a chance for physiological pulp regeneration. The approach triggering the migration and regenerative potentials of endogenous dental stem cells seemed to be a promising choice for clinical translation of pulp regeneration.^6^ The pulp regeneration based on endogenous stem cells undergoes stem cell migration, proliferation, and odontogenic differentiation. The oriented migration and differentiation of quiescent stem cells surrounding the root apex is the key point, which can be initiated and controlled by exogenous signaling molecules.^7^ Recently, in an in situ large animal model, Wnt3a, as a developmental signal, induced neo-dentin-like tissue regeneration in the root canal.^8^ Though the dentin-pulp-like tissue produced excessive mineralization, this finding suggested the possibility of pulp regeneration through reconstructing the regenerative microenvironment with developmental signals in the root canal.

During tooth development, a cascade of molecular events including the Wnt, transforming growth factor-beta (TGF-β), and bone morphogenetic protein (BMP) pathways are precisely activated.^9^ Wnt molecules promote cell growth and osteo/odontoblastic differentiation^10^ and mediate stem cell self-renewal and tooth morphogenesis.^11,12^ Previous studies have reported that Wnt3a increased mesenchymal stem cell (MSC) proliferation and decreased apoptosis^13^ and modulated the tertiary dentin formation.^14^ Compared with Wnt3a, Wnt5a may exert different roles in different tissue-derived cells. One research has reported that wnt5a negatively regulated dental papilla cell proliferation and migration.^15^ But another research has reported that Wnt5a negatively regulated palate tissue cell proliferation but induced cell migration during palate development.^16^ Moreover, overexpression of Wnt10a decreased odontoblastic differentiation-related gene expression of dentine sialophosphoprotein (DSPP) and dentin matrix protein 1 (DMP-1).^17^ TGF-β molecules have similar bio-activities and play critical roles in epithelial-mesenchymal interactions and odontoblast maturation.^18^ TGF-β1 can promote odontoblastic differentiation by upregulating the expression of DSPP and DMP-1.^19,20^ Moreover, TGF-β1 regulates cell proliferation via Smad2/3 signaling during the tail regeneration in Xenopus tropicalis tadpoles^21^ and promotes the viability and proliferation of bone marrow-derived MSCs.^22^ And TGF-β1 and TGF-β2 both induced the synthesis of the collagen matrix in pulp fibroblasts.^20^ The BMPs play an important role in cell migration and angiogenesis,^23,24^ and mediate early tooth morphogenesis and mineralization.^25^ BMP2 and BMP4 display similar potential for osteo/odontogenic differentiation.^26^ But intense inflammation, ectopic bone formation, osteoclast-mediated bone resorption, and inappropriate adipogenesis have been reported in the high-dose application of BMP2.^27,28^ BMP7 promoted the survival and proliferation of kidney cells and showed great potential for kidney tissue engineering.^29^ Meanwhile, BMP7 promoted proliferation and tube formation in human umbilical vein endothelial cells (HUVECs).^30,31^ Though these molecules share the potential on influencing the proliferation, migration, and differentiation abilities in many types of cells,^32^ the functions of inducing the endogenous stem cell have not been described during pulp regeneration. In light of these foundations of knowledge, we consider Wnt3a, TGF-β1, and BMP7 as candidate molecules for endogenous pulp regeneration.

A suitable scaffold is necessary to facilitate the migration, residence, survival, and function of stem cells. Hydrogels are injectable and biodegradable scaffolds that undergo sol-to-gel transition. Signaling molecules can be incorporated into the hydrogel during its sol-to-gel transition, which serves as an excellent platform for the 3D culture of stem cells, as well as sustained-release delivery of growth factors.^33^ Nowadays, hydrogels are broadly used to mimic the regenerative microenvironment during pulp regeneration. Type I collagen is the main component of the natural extracellular matrix (ECM) in the pulp tissue. Collagen gel supported the survival and odontogenic differentiation of stem cells from human exfoliated deciduous teeth (SHED).^34^ The transplantation of collagen gel with dental pulp stem cells (DPSCs) in dentin slices produced pulp-like tissue.^35^ Matrigel is derived from the ECM of mouse sarcoma. DPSCs and adipose tissue-derived microvascular fragments mixed with matrigel promoted pulp-like tissue regeneration in tooth root segments (RSs).^36^ Furthermore, gelatin methacryloyl (GelMA) hydrogel supports cell encapsulation and adjustable physical properties, contributing to the construction of biomimetic 3D tooth bud models and delivery of DPSCs and HUVECs for pulp regeneration.^37,38^ However, there is no clear conclusion about which hydrogel is more suitable for endogenous pulp regeneration.^39^

In the present study, with a motivation to explore an effective approach for the clinical translation of endogenous pulp regeneration, we investigated the in vitro effects of Wnt3a, TGF-β1, and BMP7 on proliferation, migration, and osteo/odontogenic differentiation capacities of h-DPSCs and angiogenesis potential of HUVECs, as well as the role of collagen gel, matrigel, GelMA in mediating the morphology and viability of h-DPSCs in 2D or 3D culture systems. Furthermore, the injectable constructs of collagen gel loading BMP7 molecule were implanted in an ectopic nude mouse model and an in situ beagle dog model to investigate the possibility of pulp regeneration. These results provide a basis for clinical translation of the endogenous pulp regeneration, suggesting a promising aspect of the future therapeutic application of the approach based on collagen gel loading BMP7 in the REPs.

## Results

### Endogenous stem cells reside in the dental pulp and apical tissues

**Fig. 1:**
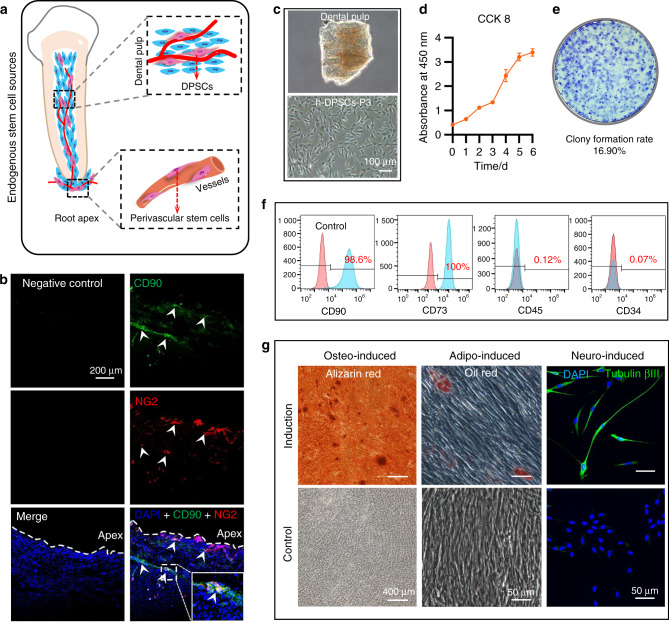
Identification of the stem cell sources. **a** The schematic diagram of the stem cells residing in the dental pulp and root apex. **b** Double-immunofluorescent labeling CD90 (MSCs) and NG2 (pericytes) in the apical tissue. Both CD90-positive and NG2-positive regions were highlighted by white arrows and enlarged in the white rectangle frame. **c** h-DPSCs isolated from dental pulp tissue. **d** The evaluation of h-DPSCs proliferation by CCK8 assay under 10% FBS/α-MEM culture. **e** The colony formation assay to evaluate the self-renewal of h-DPSCs. **f** The flow cytometry to detect MSCs markers (CD90 and CD73) expression. **g** The in vitro osteogenic, adipogenic, and neural differentiation potential of h-DPSCs evaluated by alizarin red staining, oil red staining, and immunofluorescent labeling tubulin βIII respectively. Scale bar: **b** 200 μm, **c** 100 μm, **g** 400 μm (alizarin red), and 50 μm (oil red and tubulin βIII labeled)

Firstly, endogenous stem cell sources were identified in the dental pulp and apical tissues (Fig. 1a). We obtained the pulp tissue that resides in the root canal and root apex (Supplementary Fig. S1). Immunofluorescence confirmed that some regions (white arrows) co-expressed MSCs marker CD90 (green) and pericyte marker NG2 (red)^40^ (Fig. 1b).

We then isolated h-DPSCs from dental pulp tissue (Fig. 1c). The isolated cells shared excellent proliferation and self-renewal abilities in vitro (Fig. 1d, e). Flow cytometry confirmed that these cells positively expressed MSC markers CD90 (98.6%) and CD73 (100%), while negatively expressed CD45 (0.12%) and CD34 (0.07%) (Fig. 1f). Moreover, alizarin red staining, oil red staining, and immunofluorescence of tubulin βIII showed these cells could differentiate into osteoblasts, adipocytes, and neurons after being cultured with different differentiation mediums, indicating the multi-differentiation potency of h-DPSCs (Fig. 1g).

These results confirmed that adult dental pulp tissue maintains a population of perivascular stem cells. After removing the inflammatory dental pulp tissue in the RCT, these stem cells residing around the dental apical foramen were expected to serve as the cell sources of the endogenous pulp regeneration.

### Effects of Wnt3a, TGF-β1, and BMP7 on regenerative potentials of h-DPSCs

**Fig. 2:**
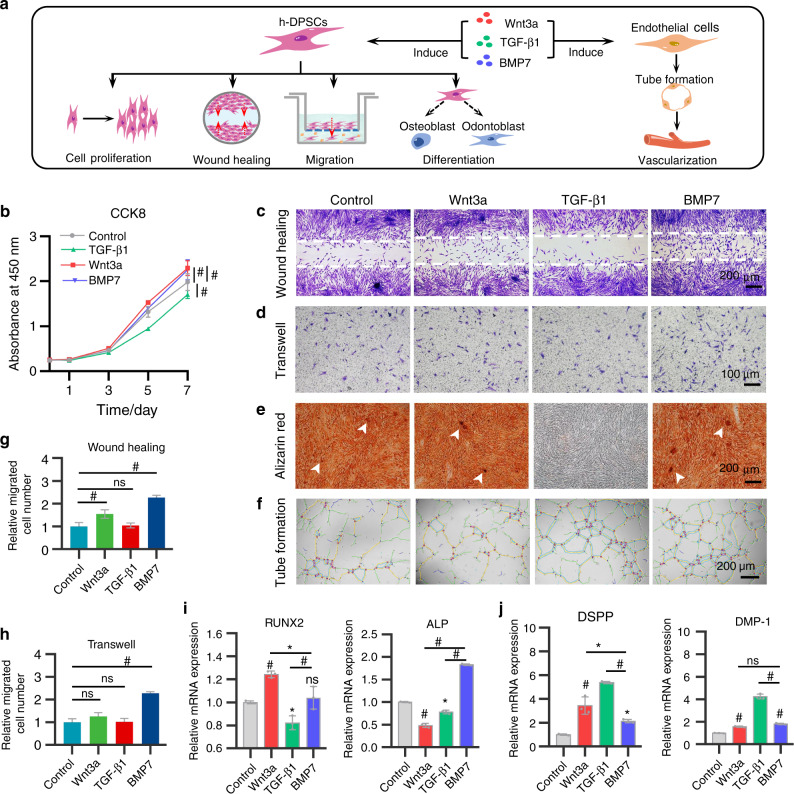
The in vitro effects of Wnt3a (50 ng·mL^−1^), TGF-β1 (10 ng·mL^−1^), and BMP7 (50 ng·mL^−1^) on h-DPSCs and HUVECs. **a** The schematic diagram of the detections of proliferation, migration, and osteo/odontogenic differentiation potentials in h-DPSCs and tube formation in HUVECs under Wnt3a, TGF-β1, and BMP7 treatment. **b** The proliferation of h-DPSCs measured by CCK8 assay on days 0, 1, 3, 5, and 7. **c**, **d** Migration of h-DPSCs detected by wound-healing assay and transwell assay after 24 h culture. **e** Alizarin red staining of calcified nodules (white arrows) in h-DPSCs cultured with osteogenic medium contained with or without Wnt3a, TGF-β1, and BMP7 for 21 days. **f** Tube formation assay to evaluate the angiogenic potential of HUVECs under Wnt3a, TGF-β1, and BMP7 treatment for 4 h. **g**, **h** Quantification of the relative invasion and migration cell number in **c** and **d**. **i**, **j** The gene expression of osteo/odontogenic markers in h-DPSCs after 3 days of culture. *P < 0.05, ^#^*P* < 0.01. NS, no significance vs Control. Scale bar: **d** 100 μm, **c**, **e**, and **f** 200 μm

To reactivate specific signals to trigger dental pulp regeneration, we focused on Wnt, TGF-β, and BMP signaling molecules, which are essential in tooth development and morphogenesis. Wnt3a mediates the dentin and root formation and maintains the self-renewal and odontogenic differentiation of stem cells. TGF-β1 is critical for epithelial-mesenchymal interactions, controlling odontoblast maturation, and promoting dentin formation. BMP7 initiates the tooth mineralization and odontogenic differentiation of DPSCs. Given the possible potential of these pivotal signals on pulp regeneration, therefore, we selected Wnt3a, TGF-β1, and BMP7 as candidate cytokines. Comprehensive evaluations of their effects on the proliferation, migration, and osteo/odontogenic differentiation capacities of h-DPSCs and tube formation of HUVECs were performed to screen suitable signaling molecules (Fig. 2a).

In the proliferation assay, we found that h-DPSCs proliferation slightly increased on day 7 in the BMP7 group and Wnt3a group, while decreasing in the TGF-β1 group from day 3 to day 7 compared to the control group (Fig. 2b).

In the wound-healing assay and transwell assay, we found that the BMP7 treatment significantly promoted the migration of h-DPSCs compared to the other three groups, while the cell migration in the Wnt3a group and TGF-β1 group were comparative to the control group (Fig. 2c, d, g, h).

The odontogenic differentiation of h-DPSCs is important for functional pulp regeneration. Our results indicated that the TGF-β1 group completely inhibited calcified nodules formation. Although quantitative analysis of the alizarin red in the Wnt3a and BMP7 groups were comparable to the control group, we found that h-DPSCs generated more calcified nodules with deeper staining (white arrowheads) after treatment of Wnt3a and BMP7 (Fig. 2e and Supplementary Fig. S2). In addition, real-time polymerase chain reaction (RT-PCR) results demonstrated that compared to the control group, runt-related transcription factor 2 (RUNX2) gene expression was upregulated in the Wnt3a group and alkaline phosphatase (ALP) gene expression was upregulated in the BMP7 group, while both RUNX2 and ALP gene expressions declined in the TGF-β1 group after 3 days of induction (Fig. 2i). Moreover, we analyzed the effect of these factors on the differentiation of h-DPSCs into odontoblasts. The expressions of DSPP and DMP-1, two markers of odontoblasts, were upregulated in all the treatment groups than in the control group (Fig. 2j).

### Effects of Wnt3a, TGF-β1, and BMP7 on tube formation of HUVECs

Remarkably, the dental pulp is surrounded by the unique anatomy of rigid dentin walls. A few main vessels grow into the root canal through the apical foramen to supply the pulp tissue. Micro-vasculature in the pulp plays a major role in maintaining the self-renew of cells and the vitality of tissues via transporting nutrients and removing the metabolites.^41^ By supplying necessary oxygen and nutrients, vasculogenesis facilitates cell survival and tissue regeneration in the tissue-engineered pulp tissue.^36,42,43^ Given that neovascularization plays an essential role in supporting stem cell migration and self-renew, we investigated the effects of these developmental signaling molecules on the angiogenic potential in HUVECs. In the tube formation assay, we observed more vessel-like network formation in the TGF-β1 and BMP7 group compared to the control group and Wnt3a group after 4 h of induction (Fig. 2f). The quantification of total branching length and the number of junctions indicated that superior to the Wnt3a treatment, the TGF-β1 and BMP7 potently mediated the angiogenic potential of HUVECs in vitro (Supplementary Fig. S3a, b). Overall, these results demonstrated that BMP7 is superior to the Wnt3a, TGF-β1 to serve as a potential signal to trigger the regenerative capacities of stem cells and revascularization for pulp regeneration.

### The dose-dependent effects of BMP7 on h-DPSCs and HUVECs

**Fig. 3:**
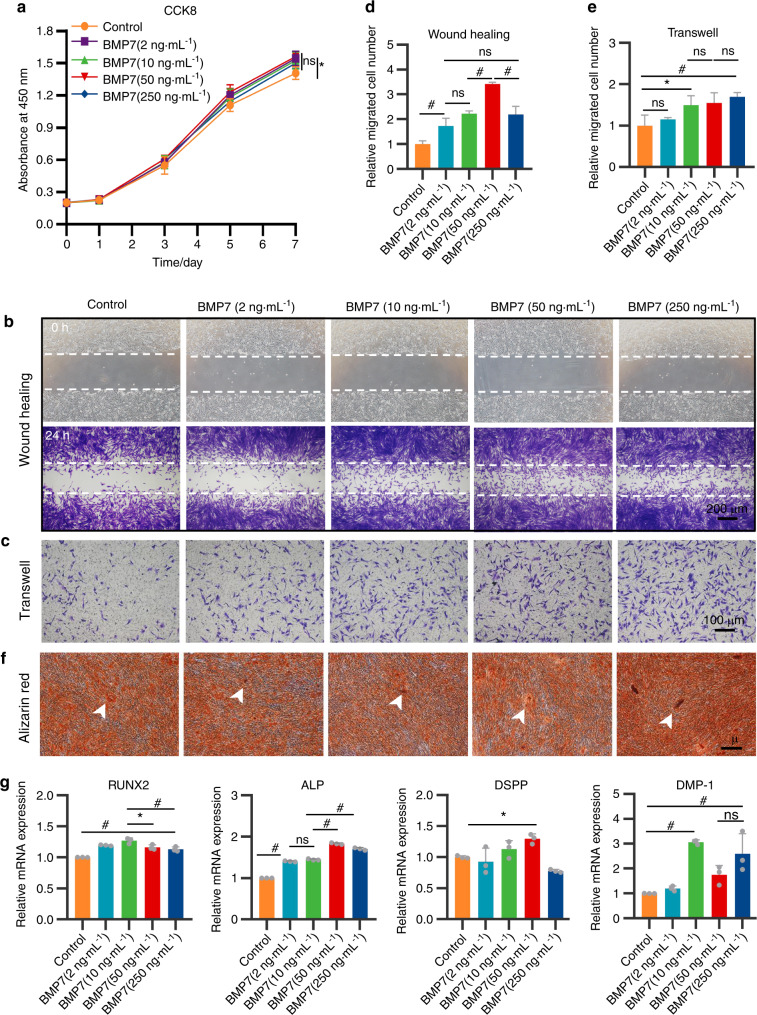
h-DPSCs treated with a range of BMP7 concentrations at 2, 10, 50, and 250 ng·mL^−1^. **a** CCK8 assay for the evaluation of h-DPSCs proliferation. **b**, **c** The wound-healing assay and transwell assay for evaluation of h-DPSCs migration ability. **d**, **e** Quantification of the relative invasion and migration cell number in **b** and **c**. **f** Alizarin red staining of h-DPSCs induced by osteogenic medium contained with or without BMP7 (2, 10, 50, and 250 ng·mL^−1^) for 21 days. **g** The expression of osteo/odontogenic markers of h-DPSCs after 3 days of culture. *P < 0.05, ^#^*P* < 0.01 vs Control. NS: no significance. Scale bar: **b** 200 μm, **c** 100 μm, **f** 500 μm

To investigate the dosage effects of BMP7 on h-DPSCs and HUVECs, we tested a range of BMP7 concentrations at 2, 10, 50, and 250 ng·mL^−1^. Cell counting kit-8 (CCK8) assay revealed that 10 and 50 ng·mL^−1^ BMP7 mildly promoted the proliferation of h-DPSCs than the control. But there was no statistical difference among the BMP7 treatment groups (Fig. 3a). The wound-healing assay demonstrated that the 50 ng·mL^−1^ BMP7 promoted more invasion of h-DPSCs than the other groups, while there was no statistical difference among BMP7 2, 10, and 250 ng·mL^−1^ groups (Fig. 3b, d). Transwell assay indicated that the BMP7 50 and 250 ng·mL^−1^ groups all promoted h-DPSCs migration than the control group (Fig. 3c), the quantitative analysis showed that the number of migration h-DPSCs increased as the BMP7 dosage increased (Fig. 3e).

Furthermore, indicated by alizarin red staining, the size and number of the calcified nodules increased as the dosage of BMP7 rose (Fig. 3f). The quantification of staining density indicated that the 250 ng·mL^−1^ BMP7 group was higher than the other groups (Supplementary Fig. S4). The RUNX2 and ALP gene was upregulated along with the BMP7 dose increased relative to the control group, and the expressions of RUNX2 in the 10 ng·mL^−1^ BMP7 group and ALP in the 50 ng·mL^−1^ BMP7 group were respectively higher than the other four groups (Fig. 3g). The DSPP gene expression was increased in the 50 ng·mL^−1^ BMP7 group, while DMP-1 gene expression was increased in the 10, 50, and 250 ng·mL^−1^ BMP7 groups (Fig. 3g).

**Fig. 4:**
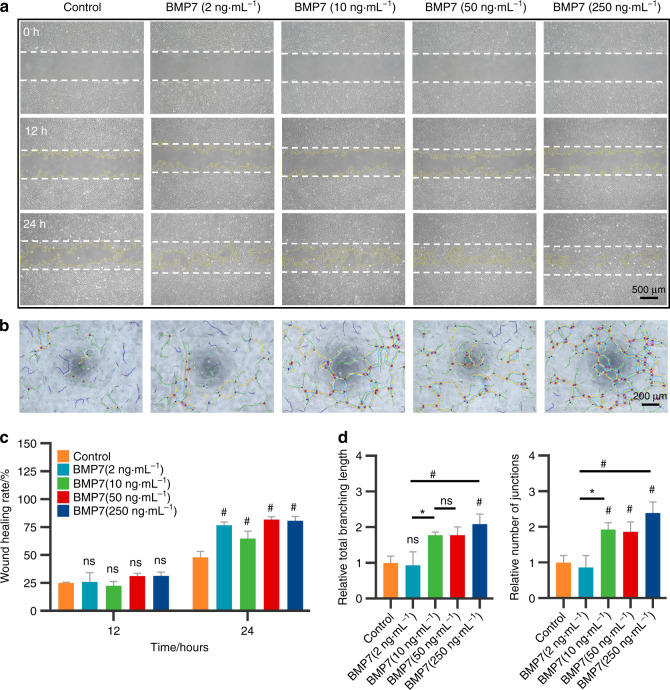
The effects of BMP7 on angiogenic potential of HUVECs. **a** The wound-healing assay to evaluate the migration of HUVECs induced by BMP7 for 12 and 24 h. **b** Angiogenic potential of HUVECs evaluated by tube formation assay after 4 h culture. **c** Quantitative analysis of the relative recovered area at 12 and 24 h in **a**. **d** Quantitative analysis of the total branching length and number of junctions formed by HUVECs in **b**. *P < 0.05, ^#^*P* < 0.01. NS: no significance. Scale bar: **a** 500 μm, **b** 200 μm

We assessed the dosage effects of BMP7 on the migration and tube formation of HUVECs. As shown in Fig. 4a, the HUVECs migrated toward the wound region after 12 h culture. After 24 h culture, more HUVECs migrated toward the wound region in the 50 and 250 ng·mL^−1^ BMP7 groups than in the other three groups. Quantitative analysis also indicated that the 50 and 250 ng·mL^−1^ BMP7 groups potently promoted migration ability in HUVECs (Fig. 4c). Tube formation assay revealed that 10, 50, and 250 ng·mL^−1^ BMP7 promoted the capillary-like structure formation in HUVECs (Fig. 4b). Quantification of the total branching length and the number of junctions showed that 10, 50, and 250 ng·mL^−1^ BMP7 apparently promoted the tube formation in HUVECs compared with the control group and 2 ng·mL^−1^ BMP7 group (Fig. 4d).

### The morphology and viability of h-DPSCs in collagen gel, matrigel, and GelMA

**Fig. 5:**
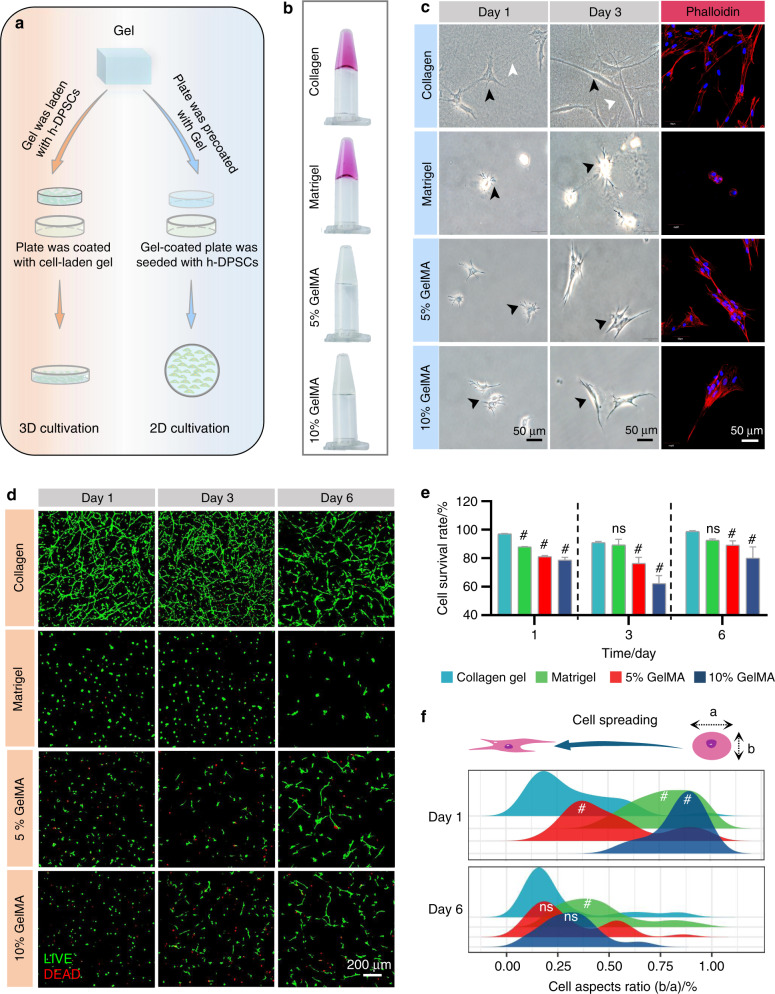
The morphology and viability of h-DPSCs in 2D and 3D culture systems. **a** Schematic diagram of the 2D and 3D culture with h-DPSCs in hydrogels. **b** Fabrication of the collagen gel, matrigel, 5% GelMA, 10% GelMA. **c** The optical pictures and phalloidin-labeled immunofluorescence pictures took from 2D cultured h-DPSCs on the surface of collagen gel, matrigel, 5% GelMA, and 10% GelMA on day 1 and day 3. **d** Live/dead staining pictures of 3D cultured h-DPSCs embedded into collagen gel, matrigel, 5% GelMA, 10% GelMA at days 1, 3, and 6. **e** Quantification analysis of the cell survival rate (live cells/dead cells). **f** The distribution analysis of cell aspect ratio (b/a) of h-DPSCs in collagen gel, matrigel, 5% GelMA, and 10% GelMA on day 1 and day 6 in **d**. *P < 0.05, ^#^*P* < 0.01 vs collagen group. NS, no significance. Scale bar: **c** 50 μm, **d** 200 μm

We fabricated 2D and 3D culture models with collagen gel, matrigel, 5% GelMA, and 10% GelMA to investigate the morphology and viability of h-DPSCs (Fig. 5a, b). Under 2D culture conditions, h-DPSCs presented a uniform spindle-shaped appearance (black arrowheads) with straightened collagen filaments (white arrowheads) in the collagen group on day 1 and day 3. On the contrary, poor cell spreading was observed in the matrigel, 5%, and 10% GelMA groups. In addition, immunofluorescence revealed that h-DPSCs labeled by phalloidin (red) evenly adhered on the surface of collagen gel, while aggregated into cell clumps on the surface of the matrigel, 5% and 10% GelMA (Fig. 5c). Under the 3D culture system, the collagen and matrigel groups showed a higher cell survival rate relative to the 5% and 10% GelMA groups on days 1, 3, and 6, though the survival rates of the 5% and 10% GelMA groups tended to rise from day 3 to day 6 (Fig. 5d, e). In addition, the h-DPSCs presented uniform and stretched morphology in the collagen gel from day 1 to day 6 (Fig. 5d). The distribution of the aspect ratios(b/a) of h-DPSCs showed that the cell spreading in the collagen group is patently better than in the other groups on day 1. On day 6, the cell spreading in the 5% and 10% GelMA groups was superior to that on day 1, which was comparative to the collagen group. However, the cell spreading was lower in the matrigel group than that in the other three groups on day 1 and day 6 (Fig. 5f).

In brief, we argued that collagen gel, which is superior to matrigel and GelMA, performed better properties in supporting cell adhesion, cell spreading, and viability of h-DPSCs.

### BMP7 mediated the pulp-like tissue ingrowth via inducing stem cells migration

**Fig. 6:**
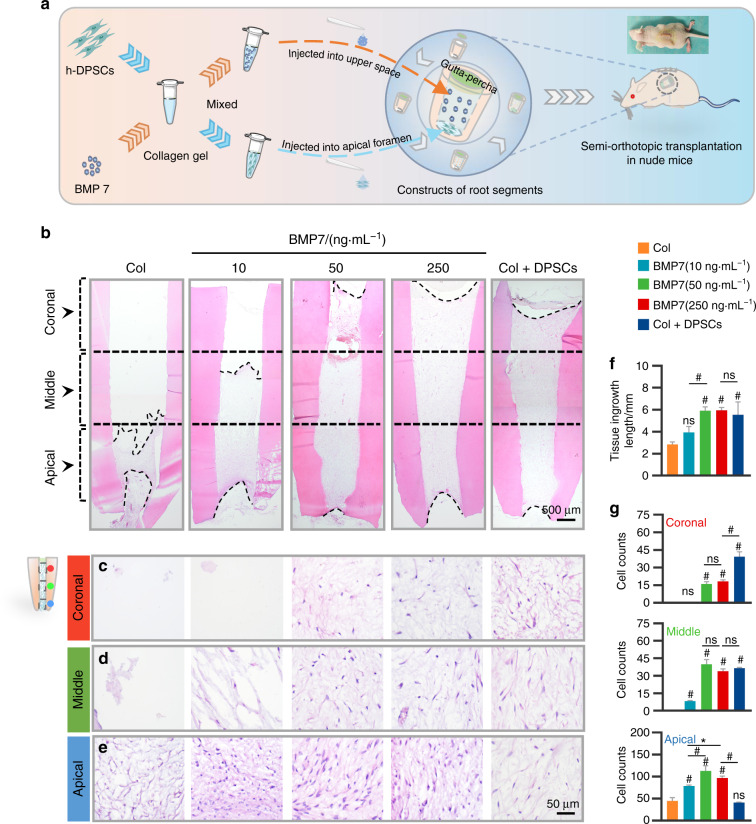
The evaluation of ingrowth of pulp tissue in the nude mice model. **a** Schematic diagram of the ectopic pulp regeneration model in nude mice. Collagen gel pre-mixed with h-DPSCs or BMP7 was respectively injected into the upper space and the apical foramen of the RSs, which were subcutaneously transplanted into the back of nude mice for 6 weeks. **b** The overall view of pulp-like tissue regeneration in the RSs by H&E staining. The distribution of the migrated cells was evaluated in the coronal (**c**), middle (**d**), and apical (**e**) regions in the root canal. **f** Quantification of the tissue ingrowth length in **b**. **g** Quantification of the average cell number per field in the coronal, middle, and apical regions. *P < 0.05, ^#^*P* < 0.01 vs the Col group. NS, no significance. Scale bar: **b** 500 μm, **c**–**e** 50 μm

Our in vitro results concluded that BMP7 mediated the migration and odontogenic differentiation of h-DPSCs and tube formation of HUVECs via a dose-dependent manner. The BMP7 concentration at 10, 50, and 250 ng·mL^−1^ might exert efficacy on pulp regeneration. Moreover, collagen gel supported the adhesion and viability of h-DPSCs. Therefore, we further investigated whether the BMP7 molecule and collagen gel can achieve pulp regeneration using an ectopic dental pulp regeneration model.^44,45^ Collagen gel supplemented with or without h-DPSCs or BMP7 was injected into the canal chambers of the RSs with a gutta-percha sealed crown and an opened apex, then 5.0 × 10^5^ h-DPSCs suspending in 10 uL collagen gel solution were implanted into the apex. Then the constructs were subcutaneously transplanted into the back of the nude mice (Fig. 6a). The Col+DPSCs group is set as a positive control.

The transplants were harvested for histological analysis after 6 weeks. Histological analysis indicated that the mean length of regenerated pulp-like tissue was less than 3 mm in the collagen group, about 4 mm in the 10 ng·mL^−1^ BMP7 group, and nearly 6 mm in the 50 and 250 ng·mL^−1^ BMP7 groups, which is comparative to the Col+DPSCs group (Fig. 6f). Notably, in the 50 and 250 ng·mL^−1^ BMP7 groups, newly formed pulp-like tissue filled in the full-length root canal, while the structure of the regenerated tissue was similar to that of the Col+DPSCs group (Fig. 6b). Besides, the cell density at the apical, middle, and coronal sites of the canal was evaluated respectively. In the apical region, all groups can observe cells, while the cell density in the 50 and 250 ng·mL^−1^ BMP7 groups was higher than that in the collagen and Col+DPSCs groups (Fig. 6e). In the middle region, the collagen group failed to induce cell ingrowth. The cell density showed no difference among the 50 and 250 ng·mL^−1^ BMP7 groups, and Col+DPSCs group, but it was less than that in the apical region (Fig. 6d). In the coronal region, no cell was observed in both the collagen group and the 10 ng·mL^−1^ BMP7 group, while the cell density in the 50 and 250 ng·mL^−1^ BMP7 groups was lower than in the Col+DPSCs group (Fig. 6c). Quantitative analysis showed that the Col+DPSCs group produced a uniform cell distribution in the entire root canal, while the other four groups presented a gradually decreased cell density from the apical region to the coronal region (Fig. 6g).

To determine whether the regenerated pulp-like tissue is host-derived or donor-derived, we further detected the human mitochondria expression in the regenerated pulp-like tissue. The immunohistochemical and immunofluorescent analysis all showed the positive expression of human mitochondria in part of the migrated cells. Moreover, the proportion of positive cells increased as the dose of BMP7 increased (Supplementary Fig. S5a, b).

### BMP7 mediated odontoblastic differentiation of the migrated h-DPSCs

**Fig. 7:**
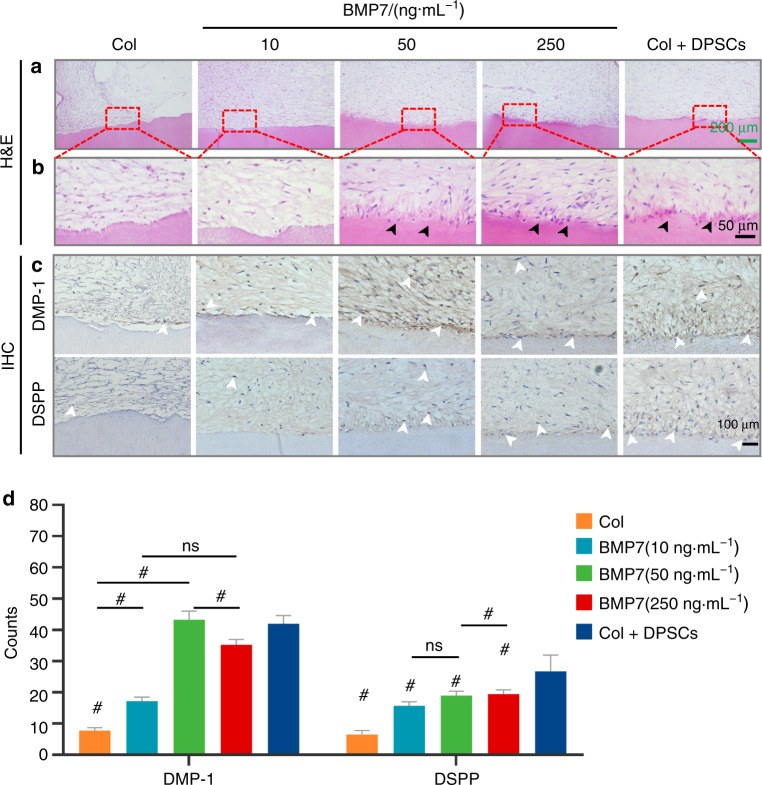
The evaluation of the dentin-pulp-like tissue regeneration in vivo by H&E and IHC. **a**, **b** The regenerated dentin-pulp-like tissues showed by H&E staining. **b** showed the enlarged images of the framed area in **a**. The black arrowheads showed polarized odontoblasts-like cells layer in the BMP7(50 ng·mL^−1^), BMP7(250 ng·mL^−1^), and Col + DPSCs groups. **c** IHC showed the DMP-1-positive or DSPP-positive odontoblasts-like cells (white arrowheads). **d** Quantitative analysis of the DMP-1-positive or DSPP-positive odontoblasts-like cell number. *P < 0.05. ^#^*P* < 0.01 vs. the Col group. NS, no significance. Scale bar: **a** 200 μm, **b** 50 μm, **c** 100 μm

Histologic analysis confirmed the pulp-like tissue regeneration in the transplants of each group (Fig. 7a). From the hematoxylin and eosin staining (H&E) staining, other than the collagen and 10 ng·mL^−1^ BMP7 groups, we can observe a layer of odontoblast-like cells (black arrowheads) lining along with the native dentin in the 50 and 250 ng·mL^−1^ BMP7 groups, similar to the Col+DPSCs group (Fig. 7b). Immunohistochemical analysis revealed the odontoblastic markers DSPP, DMP-1, and Nestin expression (white arrowheads) adjacent to the native dentin (Fig. 7c and Supplementary Fig. S6), indicating that the migrated DPSCs differentiated into odontoblasts cell lines. Quantification analysis demonstrated that compared with the collagen group, BMP7 treatment strongly triggered the odontogenic differentiation of h-DPSCs to the DMP-1-positive or DSPP-positive odontoblast-like cells (Fig. 7d).

### BMP7 mediated the pulp-like tissue regeneration via inducing neoangiogenesis

**Fig. 8:**
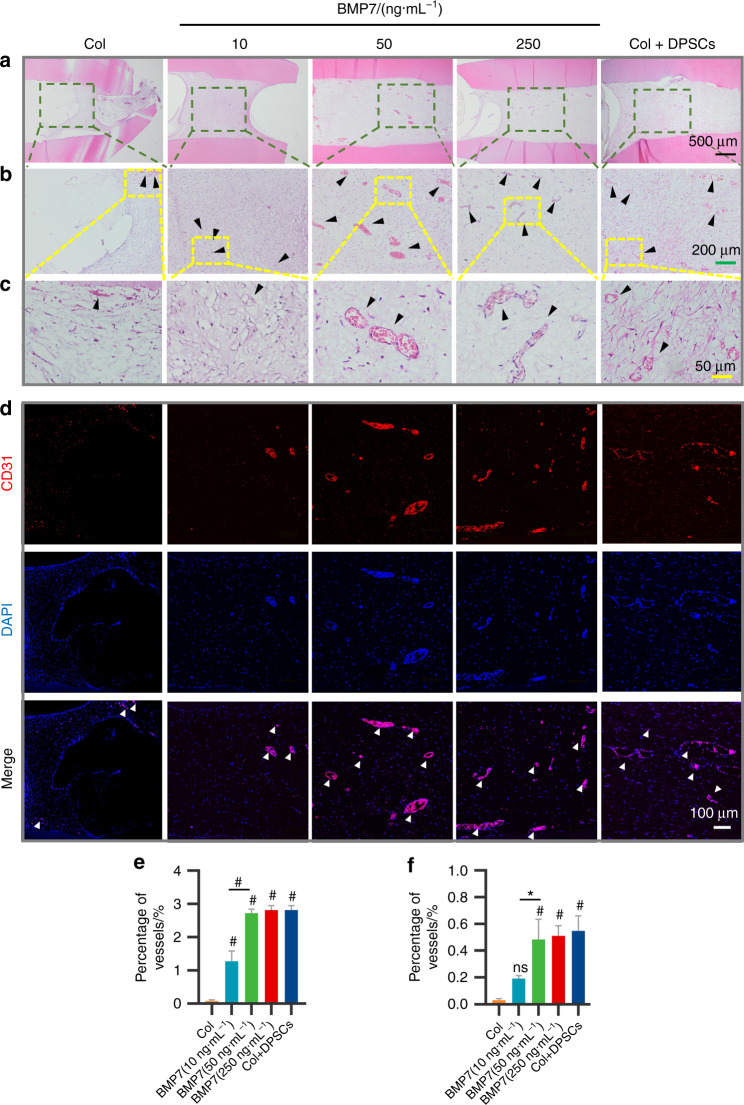
Analysis of angiogenesis in the transplants after 6 weeks of transplantation. **a** H&E staining of the selected regions of the transplants. **b** Enlarged regions of the black frames in **a**. **c** Enlarged regions of the yellow frames in **b**. The black arrowheads indicate the vessel structures in **b** and **c**. **e** Quantification of the relative areas of the vessels in **c**. **d** Immunofluorescent labeled CD31-positive vessels (white arrowheads) in the transplants. **f** Quantification of the relative areas of the vessels in **d**. *P < 0.05, ^#^*P* < 0.01 vs the Col group. NS, no significance. Scale bar: **a** 500 μm, **b** 200 μm, **c** 50 μm, **d** 100 μm

Angiogenesis is critical for tissue repair and regeneration. In vitro results indicated that BMP7 promoted vessel formation in HUVECs. Therefore, we evaluated the angiogenesis of the transplants. We observed vessel ingrowth from apical foramen in each group (Supplementary Fig. S7a). The Laser Doppler showed that higher blood perfusions were detected in the 50 and 250 ng·mL^−1^ BMP7 groups compared to the other three groups (Supplementary Fig. S7b). Furthermore, H&E staining showed vascularized pulp-like tissues with a higher vessel density and vessel diameter (black arrows) in the 50 and 250 ng·mL^−1^ BMP7 groups and the Col+DPSCs group. But no obvious mature vascular structure was observed in the collagen and 10 ng·mL^−1^ BMP7 groups (Fig. 8a–c). The semi-quantitative analysis indicated that BMP7, especially at 50 and 250 ng·mL^−1^, potently promoted vascularization compared with the collagen group (Fig. 8e). Similarly, Immunofluorescence confirmed more CD31-positive vascular network formation in the 50 and 250 ng·mL^−1^ BMP7 groups (Fig. 8d). Semi-quantification of the CD31-positive vessel area also indicated that 50 and 250 ng·mL^−1^ BMP7 induced more vessel formation than the collagen group and the 10 ng·mL^−1^ BMP7 group (Fig. 8f).

### In situ endogenous pulp regeneration in a beagle dog model

The applicability of the above findings was then tested in a preclinical beagle dog model to imitate the clinical environment. After general anesthesia, the immature teeth of the dogs (white arrowheads in the periapical film) were successively received pulpectomy, rinsing and hemostasis, hydrogel transplantation, and crown sealing (Supplementary Fig. S8a). All the teeth were harvested for histological analysis after 8 weeks. However, because of the leakage of the coronal sealing, the majority of implantation sites suffered from bacterial infection and no dental pulp tissues were regenerated. In these samples, we found that the transplants were replaced by inflammatory granulation-like tissues rather than pulp-dentin-like tissues. Nonetheless, in the samples with mild infections, the H&E staining showed that the 50 and 250 ng·mL^−1^ BMP7 induced more ingrowth of newly formed tissues and blood vessels in the canal space compared with the collagen group and 10 ng·mL^−1^ BMP7 group. This result was consistent with our results in the nude mouse model. In addition, a large amount of dentin-like or bone-like tissue formation (orange arrowheads) along with the native dentin was observed in the apical and middle region of the root canal in the 50 and 250 ng·mL^−1^ BMP7 groups, while the Col+d-DPSCs group produced dentin-like or bone-like tissue along with the whole root canal (Supplementary Fig. S8b).

However, though statistical analysis could not be performed within the context of inadequate samples, our in situ beagle dog model, to some extent, provides proof-of-concept evidence that the BMP7 molecule is contributed to the ingrowth of living tissue in an empty root canal during endogenous pulp regeneration.

## Discussion

The current study highlighted that there are endogenous cell sources for pulp regeneration in the adult dental pulp tissue or periapical tissue. Based on the in vitro experiments, we found that BMP7 is superior to the Wnt3a and TGF-β1 to trigger the regenerative capacities of stem cells and revascularization of ECs. Collagen gel is superior to matrigel and GelMA to support cell adhesion, cell spreading, and cell viability. We further investigated whether collagen gel carrying BMP7 can achieve endogenous pulp regeneration using an ectopic mouse model and an in situ beagle dog model. We observed that the 50 and 250 ng·mL^−1^ BMP7 groups strongly triggered the pulp-like tissue regeneration in vivo and induced more vessel formation than the collagen group and the 10 ng·mL^−1^ BMP7 groups. These results indicated that the approach based on collagen gel carrying BMP7 might exert a potent possibility of clinical therapeutic application for pulp loss.

There are types of quiescent stem cells including DPSCs, stem cells of the apical papilla, and periodontal ligament stem cells located in the dental pulp, apical papilla, and periodontal ligament.^46^ In our study, we identified DPSCs residing in the dental pulp tissue and perivascular stem cells niche co-expressed CD90 and NG2 in the root apex region, which provides convincing evidence of endogenous stem cell sources.

In our study, we observed a strong inhibition of calcified nodule formation of DPSCs after TGF-β1 treatment, while the odontogenic differentiation genes about DSPP and DMP-1 were upregulated. As we all know, TGF-β1 has been identified to be expressed in odontoblasts and promotes odontoblastic differentiation,^20,47^, and dentinogenesis.^48^ However, the effects on osteogenic differentiation of TGF-β1 are controversial, both positive and negative effects have been reported in previous studies.^49,50^ The concentration of TGF-β1 is important. A study has reported that 0.5–1 ng·mL^−1^ TGF-β1 stimulated ALP of SHED, whereas 5–10 ng·mL^−1^ TGF-β1 suppressed ALP by differential regulation of ALK5/Smad2/3, p38, and MEK/ERK.^51^ Similarly, another research has also found that TGF-β1 (5–10 ng·mL^−1^) downregulated RUNX2 and ALP expression via ALK5/Smad2/3 signaling.^52^ These results are consistent with the phenomenon we observed. Moreover, another study has found that dental stem cells are more inclined to odontogenic differentiation than BMMSCs,^53^ which indicated that the different cell types would have different reactions to TGF-β1 treatment.

In addition, our results found that BMP7 promoted migration and odontogenic differentiation capacities of h-DPSCs and upregulated the DSPP and DMP-1 expression, while the upregulation of RUNX2 gene expression was not obvious. According to the previous studies, we found that different BMPs may exert different influences on osteogenic differentiation by mediating distinct signaling via Wnt, Notch, and PI3K/AKT/mTOR pathways.^54^ BMP2 and BMP4 have a higher correlation to the RUNX2-dependent induction of osteoblastic differentiation than BMP7.^55^ Moreover, BMP7 has been reported to induce the highest expression levels of Smad6 and Smad7, while BMP2 and BMP4 induced modest expression levels of Smad6 and/or Smad7. And BMP7 did not significantly induce Runx2 expression but increased the accumulation of minerals,^54,56^ which is consistent with our result. In addition, we found that BMP7 potently enhanced the migration capacity and slightly affected the proliferation of h-DPSCs. These results still tie well with the previous studies, wherein the adenovirus overexpressed BMP7 in DPCs upregulated the odontoblastic differentiation and maintained an equal proliferative ability to the control group.^57^

Scaffolds are critical for cell residence and drug delivery.^58^ We found that the collagen gel exerted better performance on maintaining the cell spreading and cell distribution in the 2D or 3D culture than matrigel and GelMA. That might be explained by the fact that collagen gel is a natural ECM sharing abundant cell adhesion sites.^33^ However, we did not evaluate the migration of h-DPSCs in our 3D culture experiment due to the poor effects on cell migration when cultured with pure scaffolds according to previous research by our team. Meanwhile, according to the in vitro results, we believed that BMP7 is the key factor in inducing the migration of h-DPSCs rather than the scaffolds. The scaffolds synergistically provided an ideal regenerative microenvironment and maintained cell spreading and cell viability during the pulp regeneration process.

Furthermore, after 6 weeks of transplantation in the nude mouse model, we found that 50 and 250 ng·mL^−1^ BMP7 groups induced more ingrowth of pulp-like tissue than collagen or 10 ng·mL^−1^ BMP7 groups. We observed the morphology of the migrated cells in the regenerated tissue was different. To figure out whether these cells expressing the same markers and the regenerated pulp-like tissue are host-derived or donor-derived, we conducted the human mitochondria detection using IHC and IF. We found that part of the migrated cells in the pulp-like tissue was human mitochondria positive, and the proportion of positive cells increased as the dose of BMP7 increased. Therefore, we speculated that the regenerated pulp-like tissue is partially host-derived and partially donor-derived. In addition, polarized odontoblasts-like cells expressing human DSPP, DMP-1, and Nestin were observed in the 50 and 250 ng·mL^−1^ BMP7 groups, which indicated that the migrated h-DPSCs differentiated into odontoblasts with the dentin formation potential.

We surprisingly observed abundant vessel formation in the 50 and 250 ng·mL^−1^ BMP7 groups. This result highlighted that the BMP7 signal mediated the well-vascularized and structured pulp-like tissue regeneration might be largely associated with angiogenesis, which is attributed to the BMP7 treatment according to the effects on migration and tube formation of ECs.

The in situ pulp regeneration in the beagle dog model is aimed at mimicking the regenerative environment in the clinic. Since no standard procedure can be used for endogenous pulp regeneration in large animals, considering possible issues regarding animal welfare, we preliminarily used only three beagle dogs for a pilot study. Regrettably, most sites were infected due to crown leakage. The possible reasons would include the following points. The cavity shape of pulp access is cylindrical, which lacks reliable support for the upper resin to resist external forces. To avoid the adverse effects, we abandoned the acid etching step, which potently reduces the stability of the resin. The occlusal force of the beagle dog is so strong that the coronal fillings were damaged and then leading to pulp infection. Considering such a situation, we decided to halt the further research of expanding samples. In the samples with mild infections, the H&E staining showed that the 50 and 250 ng·mL^−1^ BMP7 induced ingrowth of newly formed tissues with dentin-like or bone-like tissue formation probably produced by BMP7-induced odontoblasts or osteoblasts. Nonetheless, our dog experiment provided the basis for subsequent large-sample animal experiments and aroused the attention of reinfection issues during pulp regeneration. Therefore, we believed that the dog experiment can be considered as proof of the concept that BMP7 enables to induce endogenous pulp regeneration, and still has practical significance to some degree.

According to the previous studies, BMP7 has been found to promote cell migration via activating the classical BMP signaling pathway by upregulating the phosphorylated Smad1/5/8 levels^59,60^ and other non-classical pathways such as Smad5-p75 neurotrophin receptor (p75NTR) signaling^61^ and PI3K-dependent chemotropic signaling.^62^ On the other hand, BMP7 signaling has been found to increase EC and vascular smooth muscle cell migration through ALK2, ALK3, or ALK6 signaling^63^ and indirectly induce vascular endothelial growth factor expression.^64^ These mechanisms may partially explain the results we observed in vitro that the BMP7 molecule potently promoted the migration of DPSCs and the tube formation of HUVECs and the results in vivo that the BMP7 molecule successfully induced stem cells migration and angiogenesis in the regenerated pulp-like tissue.

## Conclusion

In summary, our study revealed the promising effects of the BMP7 molecule to serve as a chemotactic factor to trigger the dental pulp-like tissue regeneration via inducing stem cell migration and as a pro-angiogenic factor to induce angiogenesis in the regenerated pulp tissue. Collagen gel, as a regenerative microenvironment, supports cell adhesion, spreading, and viability. Remarkably, these results suggest a promising approach based on collagen gel loading BMP7 in the therapeutic application and clinical translation of endogenous pulp regeneration.

## Materials and methods

### Cell culture

All the procedures were under a protocol approved by the Ethics Committee, West China School of Stomatology, Sichuan University, China. h-DPSCs were isolated as described previously.^65^ Pulp tissue was extracted from wisdom molars of the patients aged 16–20 years who have signed the informed consent and scissored into tiny pieces before being digested with 3 mg/mL collagenase type I (Sigma-Aldrich, St Louis, MO) for 30 min at 37 °C. The digested tissue and single cells were seeded into 25 cm^2^ culture flasks (Corning, NY) with minimal essential medium-α (α-MEM; HyClone, Marlborough, MA) containing 10% fetal bovine serum (FBS; Gibco, Grand Island, NY) and 1% penicillin-streptomycin under 5% CO_2_ at 37 °C. In addition, dog dental pulp stem cells (d-DPSCs) were obtained from the canines of beagle dogs (6 months old) according to the above method. HUVECs (ATCC) were cultured with endothelial medium (Sigma, St Louis, Missouri) containing 10% horse serum (Sigma). All cells at passages 3–5 were used in the following experiments.

### Identification of endogenous stem cells sources

The isolated cells from human dental pulp were identified by flow cytometry to detect the MSC markers using the conjugated antibodies including CD73/PE (BD, Franklin Lakes, NJ), CD90/PE(BD), CD34/FITC(BD), CD45/FITC(BD). The single-cell suspension (>1 × 10^5^ cells) was incubated with 2% FBS/PBS containing the above antibodies for 30 min at 4 °C. The isotype served as the negative control. Results were detected using an Accuri C6 flow cytometer (BD) and analyzed by FlowJo V10 software (BD). Moreover, the colony-forming assay was performed to evaluate h-DPSCs self-renewal. h-DPSCs (1 × 10^3^ cells) were cultured with 10% FBS/α-MEM in 10 cm plates for 7 days. The ratio of colony formation was calculated by crystal violet (Solarbio) staining. CCK8 (KeyGEN BioTECH, Nanjing, China) was performed to evaluate the proliferation of h-DPSCs. h-DPSCs were cultured with 10% FBS in a 96-well plate (Corning, NY, USA) at 2 × 10^3^ cells per well. The proliferation was detected on days 1, 3, 5, and 7 according to the manufacturer’s instructions. The multilineage differentiation of h-DPSCs was assessed by alizarin red staining (Sigma-Aldrich), oil red staining (Sigma-Aldrich), and immunofluorescence (tubulin βIII) (Abcam, Cambridge, UK) after osteo/odontogenic, adipogenic, and neurogenic induction as described previously.^66^

Endogenous pulp regeneration relies on the migration of precursors or stem cells that resided in the residual tissue in periapical regions. These stem cells maintain vitality and stemness even though under moderately inflammatory conditions.^67^ Moreover, the periapical tissues with periapical abscess and cysts were also observed with positive expression of MSCs markers,^68^ indicating another rich source of MSCs. Furthermore, a group of DPSCs expressed the pericyte marker, indicating a phenotype consistent with perivascular cell populations.^69^ We harvested the tissue from the root apex and identified the cell phenotype via immunofluorescence. The harvested tissues were washed with PBS three times, then fixed in the ice-cold acetone for 15 min, further dehydrated in 30% sucrose in PBS. Frozen sections with 10 μm thickness were prepared to detect the expressions of MSCs marker CD90 (Zengneng, Chengdu, China) and pericytes marker NG2 (Santa Cruz, Texas, USA) according to the manufacturers’ recommendations. The pictures were captured with a magnification of ×20 using a confocal microscope (Olympus, Tokyo, Japan).

### Cell proliferation assay

The effects of different cytokines (Wnt3a (50 ng·mL^−1^, R&D Systems, USA), TGF-β1 (10 ng·mL^−1^, R&D), and BMP7 (50 ng·mL^−1^, R&D)) or BMP7 at different concentrations (2, 10, 50, and 250 ng·mL^−1^) on h-DPSCs were evaluated. Firstly, CCK8 assay was used to detect the h-DPSCs proliferation. h-DPSCs were cultured with 5% FBS/α-MEM containing the above cytokines (the treatment groups), while 5% FBS/α-MEM served as the control group. The absorbance at 450 nm was detected on days 0, 1, 3, 5, and 7 using an absorbance microplate reader (Thermo, USA).

### Detection of migration capacity in h-DPSCs

The migration capacity of h-DPSCs was evaluated by the wound-healing assay and transwell assay. Prior to each experiment, h-DPSCs were starved with a serum-free medium for 6 h. In wound-healing assay, a cell-free region was fabricated using a 1000 µL pipette tip in 6-well plates after monolayer cell sheet formation. Then h-DPSCs were incubated with either 1% FBS/α-MEM or extra supplementation of the above cytokines for 24 h. Twenty-four-well plates of inserts with an 8.0 μm pore size (Corning, USA) were used in the transwell assay. In all, 5 × 10^4^ h-DPSCs in 200 µL α-MEM were seeded into the upper chambers, while the lower chambers were added 500 µL medium mentioned above. After 24 h incubation, h-DPSCs in the upper chamber were removed with a swab. The invasion cells and migration cells stained with crystal violet were counted by ImageJ software (version 10.2; National Institutes of Health, Bethesda, MD, USA).

### Evaluation of osteo/odontogenic differentiation in h-DPSCs

To evaluate the osteo/odontogenic differentiation of h-DPSCs, the mineralization nodules were labeled by alizarin red staining after 21 days of incubation with an osteogenic medium supplemented with or without cytokines mentioned above. The staining intensity was quantified by the absorbance at 562 nm after 10% (w/v) cetylpyridinium chloride (Sigma-Aldrich) treatment for 15 min at 37 °C. Meanwhile, the gene expression of DSPP, DMP-1, RUNX2, and ALP were detected after 3 days of induction. The total RNA was extracted using a Cell/Tissue Total RNA Isolation Kit (Vazyme Biotechnology, China), and reversely transcribed into cDNA using an iScript™ cDNA Synthesis Kit (Vazyme Biotechnology, China). The relative expression of target genes was detected by RT-PCR followed by the instructions of SYBR® Green PCR Master Mix (Applied Biosystems). Glyceraldehyde-3-phosphate dehydrogenase serves as the reference gene. The primers (Sango Biotech, Shanghai, China) are listed in Supplementary Table S1.

### Evaluation of angiogenic capacity in HUVECs

The angiogenic capacity of HUVECs under different cytokines (Wnt3a (50 ng·mL^−1^), TGF-β1 (10 ng·mL^−1^), and BMP7 (50 ng·mL^−1^)) treatment was evaluated by tube formation assay. In all, 1 × 10^4^ HUVECs in 50 µL endothelial medium containing 10% horse serum with or without the mentioned cytokines were seeded into a tube formation slide (Ibidi, Germany) that was precoated with 10 µL matrigel. The pictures were captured at 4 h by an optical microscope. Results were analyzed by ImageJ.

The wound-healing assay and tube formation assay was further conducted to evaluate the effects of BMP7 at 2, 10, 50, and 250 ng·mL^−1^ on HUVECs. The pictures in the wound-healing assay were taken at 12 and 24 h.

### Morphology and viability of h-DPSCs in 2D and 3D culture systems

According to the manufacturers’ instructions, we fabricated hydrogels of the collagen gel (3 mg·mL^−1^, Nitta Gelatin, Japan), matrigel (8 mg·mL^−1^, Corning, NY, USA), and GelMA (5% and 10% of mass/volume, EFL, Suzhou, China). For 2D culture, confocal dishes were precoated with 100 µL collagen gel and matrigel solution followed by 30 min incubation at 37 °C, while precoated 100 µL GelMA solution followed by 40 s light-curing for gel transition. Then 3 × 10^4^ h-DPSCs were seeded on the surface of gels and images were captured using an optical microscope on day 1 and day 3. After phalloidine immunostaining, the cytoskeleton of h-DPSCs was observed by a confocal microscope on day 3. For 3D culture, 1 × 10^6^ h-DPSCs were resuspended into 1 mL gel solution. Cell viability was detected using a Live/Dead staining kit (KeyGEN BioTECH) on days 1, 3, and 6. Live cells with green fluorescence and dead cells with red fluorescence were identified by a confocal microscope and quantified by ImageJ. Moreover, five fields of every single image were randomly selected (the cell number of each field was about 6) to analyze the cell spreading from day 1 to day 6 by calculating the aspect ratio (the ratio between the short axis and the long axis) of h-DPSCs.

### Ectopic pulp regeneration in a nude mouse model

A nude mouse model was designed according to a previous study.^44^ All procedures were conducted under ethical guidelines and approved by the Ethics Committee, West China School of Stomatology, Sichuan University, China (WCHSIRB-D-2021-304). The healthy premolar teeth (*N* = 40) were extracted from the patients receiving orthodontic therapy with informed consent. All the teeth should be healthy and intact. Then teeth were trimmed into RSs 8–10 mm in length using fissure burs and ultrasonically cleaned with sterile PBS for 10 min, repeating three times. These RSs were subsequently treated with 17%, 10%, and 5% ethylene diamine tetraacetic acid (EDTA, KESHI) in PBS for 10 min and soaking in the 1% penicillin/streptomycin solution at 4 °C for more than 3 days. Finally, these RSs were fabricated into a gutta-percha (Xingyu, Shanghai, China) sealed crown and left with an opened apex. Moreover, the sterile saline was injected into the chambers to ensure no liquid leakage. The groups were designed as follows: (1) collagen gel (the negative control); (2) collagen gel + h-DPSCs (1.0 × 10^6^/mL, the positive control); (3) collagen gel + BMP7(10 ng·mL^−1^); (4) collagen gel + BMP7 (50 ng·mL^−1^); (5) collagen gel + BMP7 (250 ng·mL^−1^). The day before transplantation, collagen gel supplemented with or without BMP7 was injected into the canal chambers, and 5.0 × 10^5^ h-DPSCs suspended in 10 µL collagen gel solution were implanted into the root apex to stimulate endogenous stem cells. The positive group was merely filled with h-DPSCs at a density of 1.0 × 10^6^ cells per 1-mL collagen gel solution. All the constructs were incubated at 37 °C for 30 min. These RSs were implanted into the dorsal subcutaneous space of 6-week-old female *Balb/c* nude mice (GemPharmatech, Nanjing, China). All the transplants were harvested after 6 weeks, fixed with 4% paraformaldehyde for 8 h, demineralized with 17% EDTA at 37 °C for 8 weeks, and further prepared for histological analysis.

### In situ endogenous pulp regeneration in a beagle dog model

The 6-month-old beagle dogs (*N* = 3) were purchased from Ensiweier Co, Ltd (Chengdu, China). All procedures were conducted under ethical guidelines. Total 20 single-root immature anterior teeth and 15 double-root immature premolar teeth were respectively and randomly divided into the following groups: (1) collagen gel (the negative control); (2) collagen gel + d-DPSCs (the positive control); (3) collagen gel + BMP7 (10 ng·mL^−1^); (4) collagen gel + BMP7 (50 ng·mL^−1^); (5) collagen gel + BMP7 (250 ng·mL^−1^). Each group included four anterior teeth and three premolar teeth. Preoperative digital periapical films confirm the open root apex and exclude the lesions in the root canal. The surgery was performed based on a previous description.^70^ Under general anesthesia induced by Zoletil 50 (Virbac, France) 10 mg·kg^−1^ intramuscular injection, the beagle dogs successively received the pulpectomy, rinsing and hemostasis, transplantation, and sealing. Firstly, the pulp chambers were mechanically exposed using a diamond bur. the pulp tissues were removed by sterile stainless steel endodontic broaches (Dentsply, USA). Then the root canals were rinsed with 1.25% sodium hypochlorite (NaClO, Longly, Wuhan, China), 17% EDTA (Longly, Wuhan, China), and sterile saline. After complete hemostasis with sterile paper tips, the constructs were injected into the root canals. The access was filled with 1-mm-thick calcium hydroxide (Guanya, China) and 2 mm-thick resin (Dentex, China). Eight weeks later, the teeth were harvested for histological analysis.

### Blood perfusion measurement

Before the transplants were harvested, the blood flow was monitored using a Laser Doppler Flowmeter (Moor Instruments, Axminster, UK) according to the manufacturer’s specifications. The values were presented with perfusion units.

### Histological analysis

All samples were embedded in paraffin blocks and sectioned longitudinally. H&E was performed to evaluate the morphology and structure of the regenerated tissues. To evaluate the neovascularization in the transplants, three sections stained with H&E were captured using an optical microscope (Olympus) at ×4, ×10, and ×40 magnification. In addition, a red fluorescence-labeled CD31 antibody (Santa Cruz) was used to detect the ECs. Images were taken at ×20 magnification. The areas of vessels in H&E images and CD31^+^ cells-lined lumens in immunofluorescent images were respectively quantified by ImageJ. Human-specific DSPP antibody (Santa Cruz), DMP-1 antibody (Biovision, Milpitas, CA), and Nestin antibody (Santa Cruz) were detected to confirm the odontoblastic differentiation of the migrated h-DPSCs by immunohistochemistry. Human-specific mitochondria antibody (Abcam) was detected to determine whether the regenerated pulp-like tissue is host-derived or donor-derived using immunohistochemistry and immunofluorescence. The skin tissue was used for negative control.

### Statistical analysis

All experiments were repeated in triplicate. The one-way or two-way analysis of variance test was conducted for statistical analysis. All data were presented with mean values ± standard deviation using GraphPad Prism 8. Statistical significance was accepted at *P* < 0.05.

## Supplementary information

Figures S1–S8

Table S1
