# Supplementary material for: Bone morphogenetic protein 7 mediates stem cells migration and angiogenesis: therapeutic potential for endogenous pulp regeneration
Source: Int J Oral Sci. 2022 Jul 20;14:38. doi: 10.1038/s41368-022-00188-y (PMC9300630; doi:10.1038/s41368-022-00188-y)
Supplement: Supplementary file 1 — Figures S1–S8 [file 41368_2022_188_MOESM1_ESM.docx]

Supplementary materials——Figures


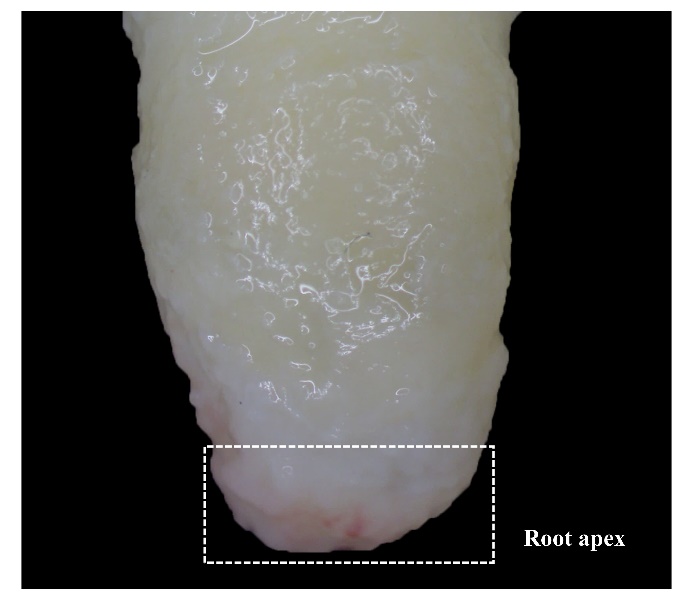


**Fig. S1** **Apical tissues obtained from the root apex.** The white frame showed the apical tissues in the the root apex.


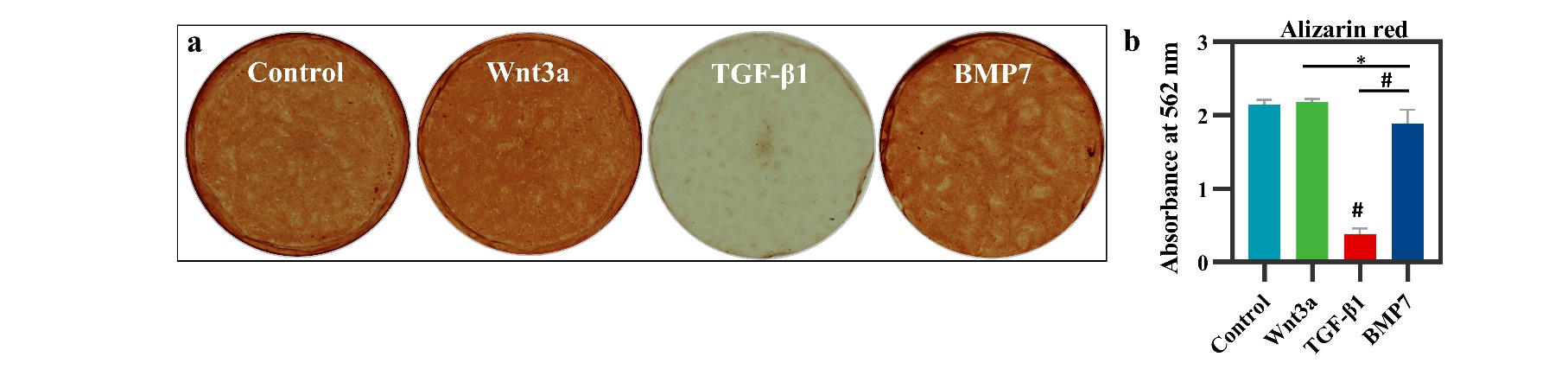


**Fig. S2** **The evaluation of alizarin red staining in h-DPSCs under Wnt3a, TGFβ-1, and BMP7 treatment.** (a) The depth of alizarin red staining in h-DPSCs cultured with osteogenic medium contained with or without Wnt3a, TGFβ-1, BMP7 for 21 days. (b) Quantitative analysis of alizarin red staining in a.


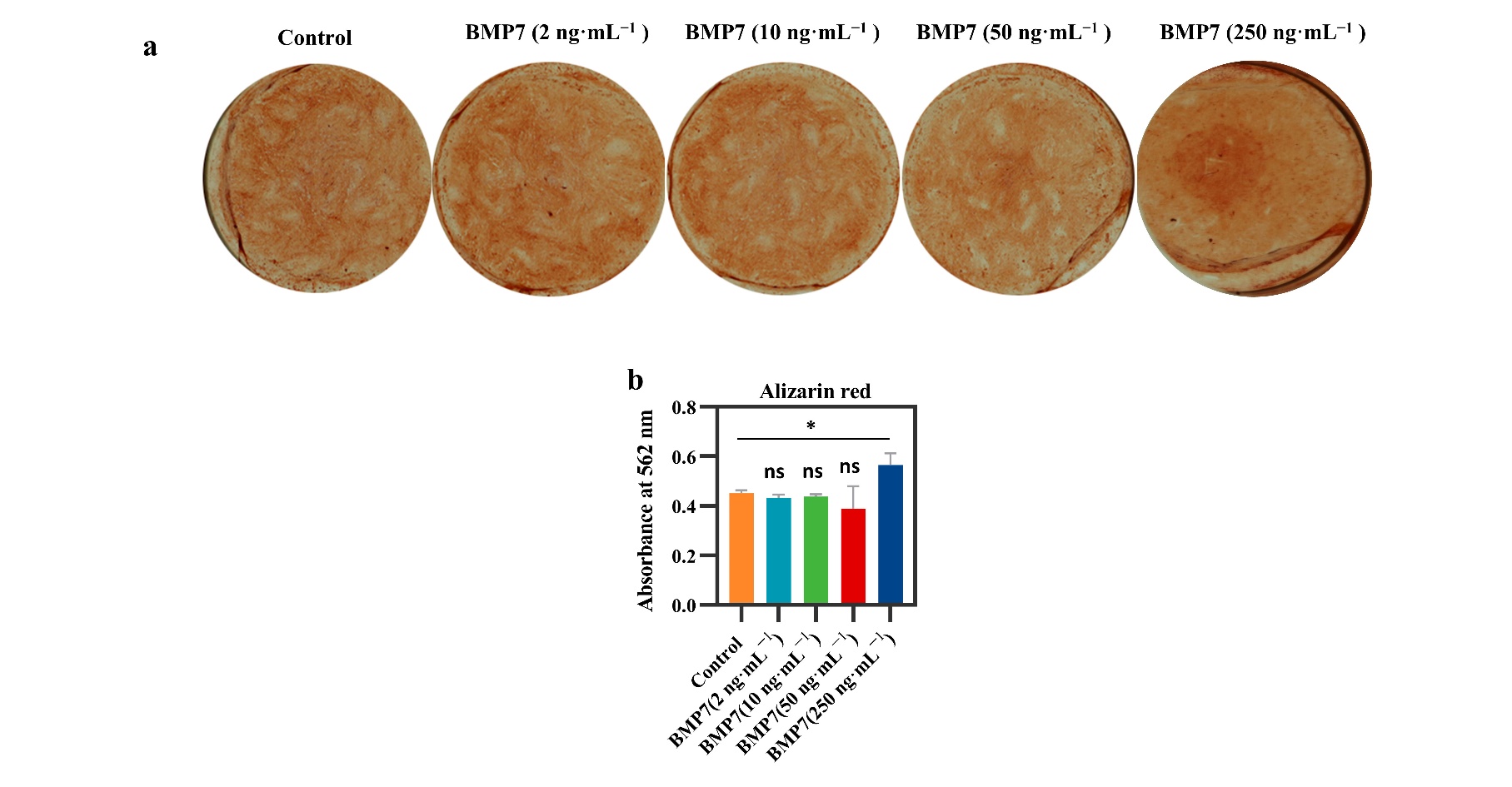


**Fig. S4** **The evaluation of alizarin red staining in h-DPSCs induced by osteogenic medium contained with or without BMP7 (2 10 50 and 250 ng·mL^−1^) for 21 days.** (a) The depth of alizarin red staining. (b) Quantitative analysis of alizarin red staining in a.


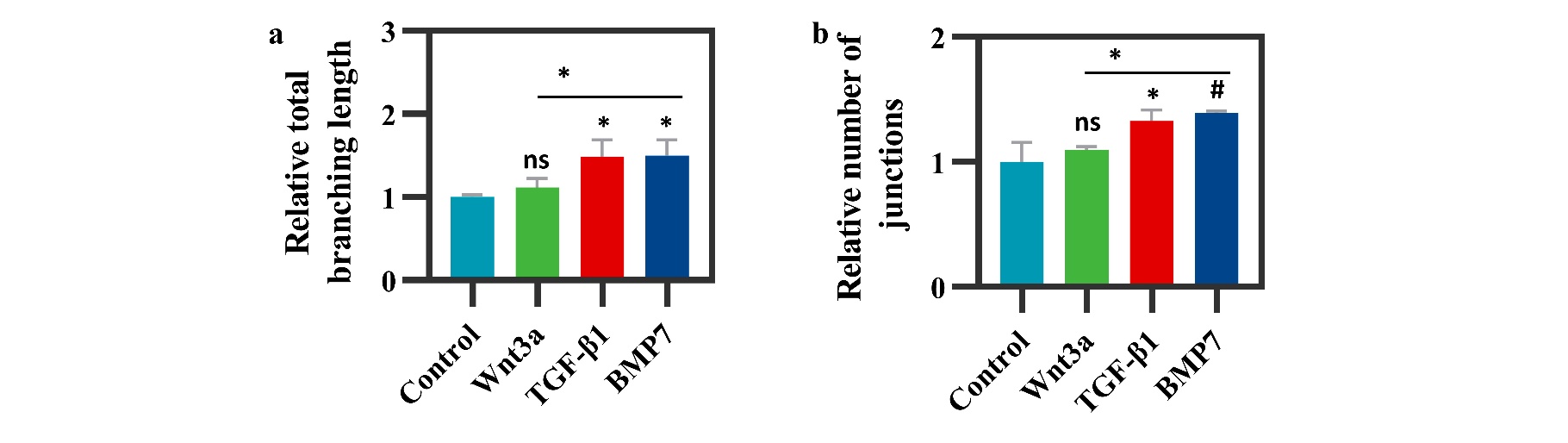


**Fig. S3** **Semi-quantitative analysis of the tube formation assay in Fig. 2f.** (a) The evaluation of total branching length. (b) The evaluation of the number of junctions.

**Fig. S5 The evaluation of human-specific mitochondria expression in the regenerated pulp-like tissue by IHC and IF.** (a) IHC showed the human mitochondria positive cells in the dental pulp-like tissue. (b) IF showed that the red-labeled human mitochondria positive cells in the dental pulp-like tissue and the proportion of positive cells increased as the dose of BMP7 increased. Scale bar: (a) 20 μm, (b) 50 μm.


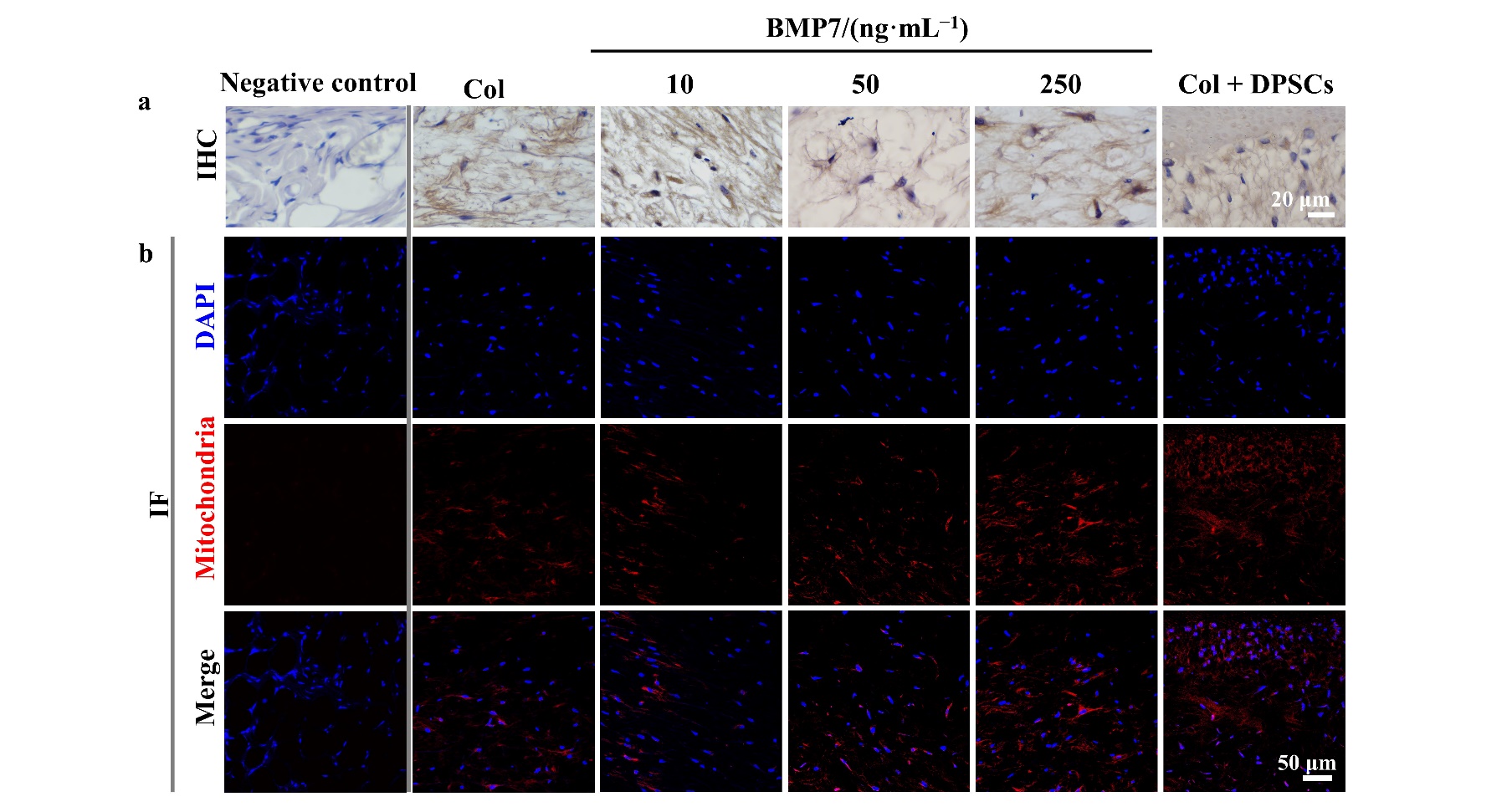

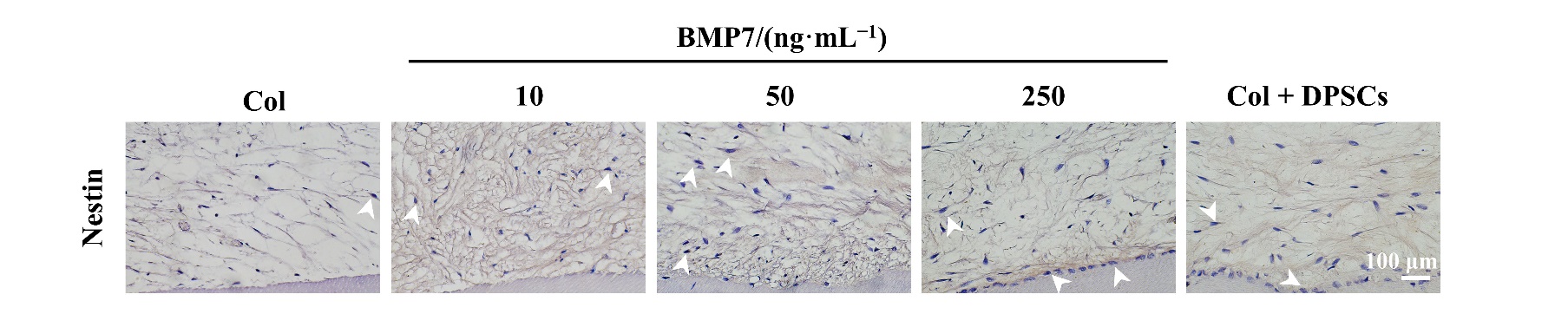


**Fig. S6 The evaluation of Nestin expression in the pulp-like tissue by IHC.** IHC showed that part of the migrated cells in the regenerated pulp-like tissue positively expressed the Nestin (white arrowheads). Scale bar: 100um.


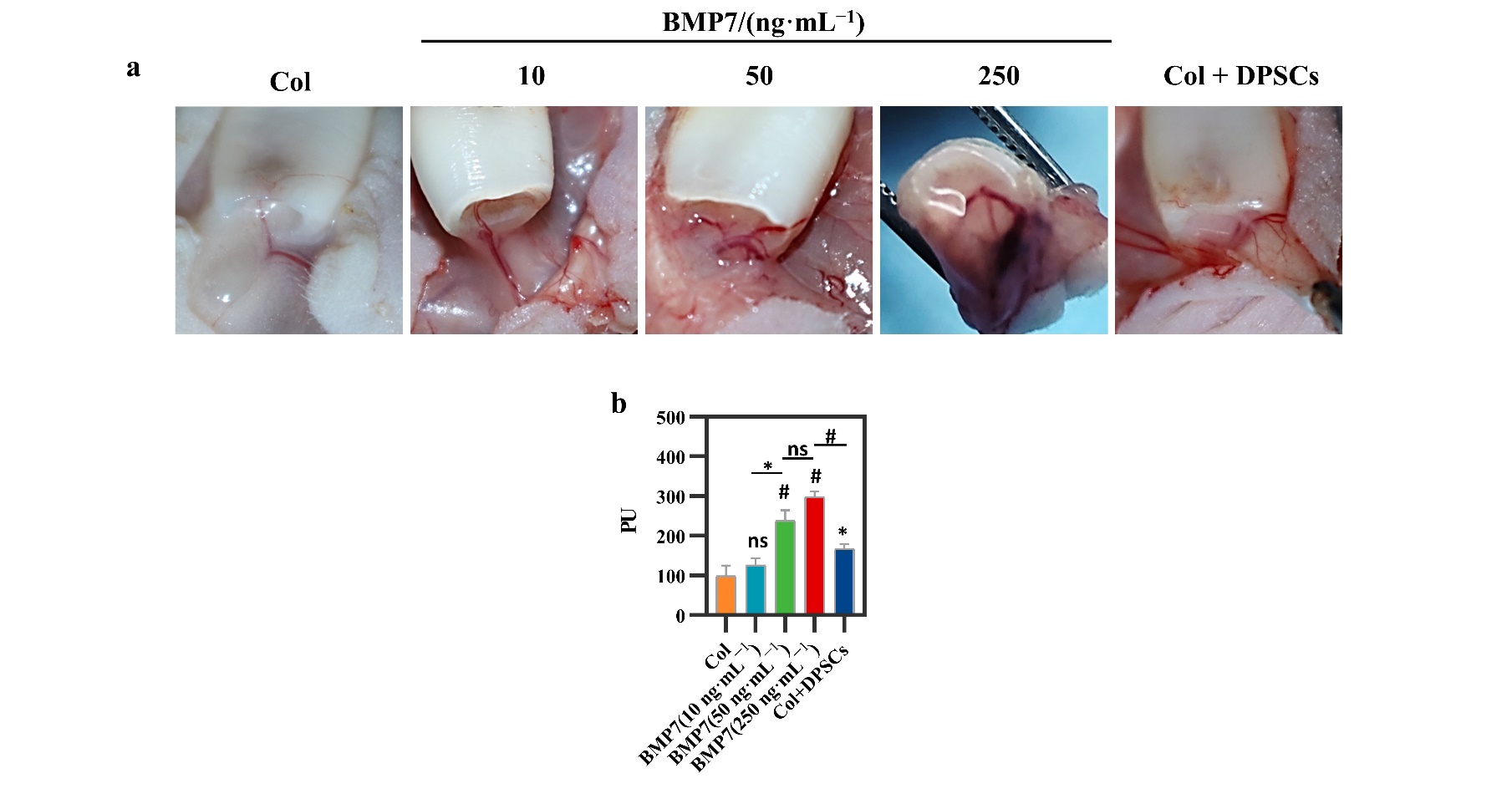


**Fig. S7 The evaluation of blood vessels ingrowth.** (a) Blood vessels ingrowth from apical foramen. (b) The measurement of blood flow perfusion by Laser Doppler Flowmetry before the transplants were harvested.


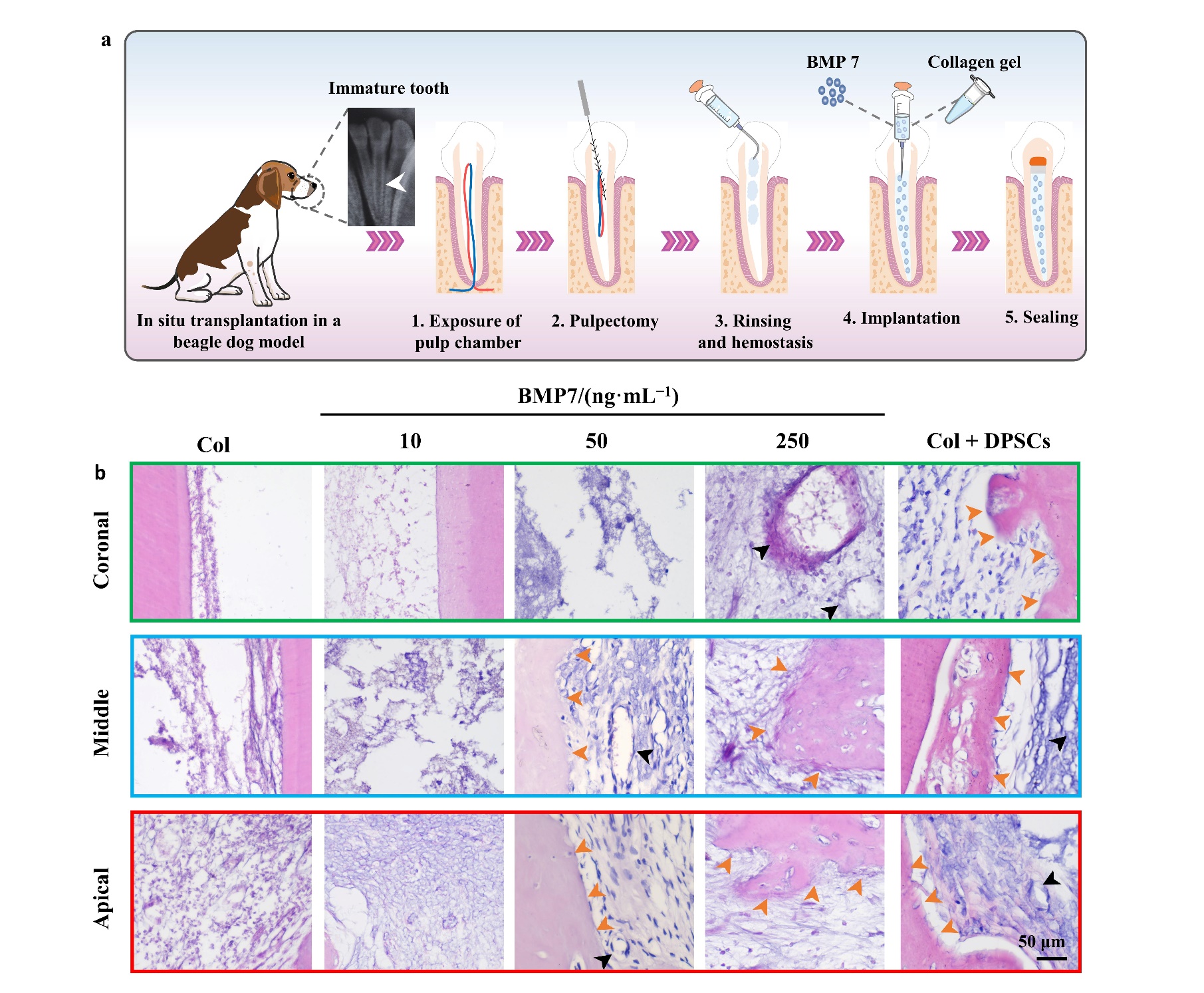


**Fig. S8** **The evaluation of the regenerated tissue ingrowth in a preclinical beagle dog model after 8 weeks transplantation.** (a) The schematic diagram of endogenous pulp regeneration in a beagle dog model. The immature teeth (white arrowheads in the periapical film) were selected and orderly received pulpectomy, rinsing and hemostasis, transplantation, and crown sealing. (b) Ingrowth of newly formed tissues in the coronal, middle, and apical regions of root canals detected by H&E staining. The orange arrowheads indicate dentin-like or bone-like tissue; Black arrowheads indicate blood vessels. Scale bar: (b) 50 μm.
