# Supplementary material for: Bone morphogenetic protein 7 mediates stem cells migration and angiogenesis: therapeutic potential for endogenous pulp regeneration
Source: Int J Oral Sci. 2022 Jul 20;14:38. doi: 10.1038/s41368-022-00188-y (PMC9300630; doi:10.1038/s41368-022-00188-y)
Supplement: Supplementary file 2 — Table S1 [file 41368_2022_188_MOESM2_ESM.docx]

| **Table S1**- Quantitative Real-time Polymerase Chain Reaction Primer Information | | |
| --- | --- | --- |
| **Genes** | Primer sequences | Length (bp) |
| **RUNX2** | F: 5’-CTTTACTTACACCCCGCCAGTC-3’  R: 5’-AGAGATATGGAGTGCTGCTGGTC-3’ | 22 |
| **ALP** | F: 5’-TAAGGACATCGCCTACCAGCTC-3’  R: 5’-TCTTCCAGGTGTCAACGAGGT-3’ | 22 |
| **DSPP** | F: 5’-CGACATAGGTCACAATGAGGATGTCG-3’  R: 5’-TTGCTTCCAGCTACTTGAGGTC-3’ | 26 |
| **DMP-1** | F: 5’-AGCATCCTGCTCATGTTCCTT-3’  R: 5’-TGATGACTCACTGCTCTCCAAG-3’ | 21 |
| **GAPDH** | F: 5’-CTTTGGTATCGTGGAAGGACTC-3’  R: 5’-GTAGAGGCAGGGATGATGTTCT-3’ | 22 |
